# A 3D Cell Culture Organ-on-a-Chip Platform With a Breathable Hemoglobin Analogue Augments and Extends Primary Human Hepatocyte Functions *in vitro*

**DOI:** 10.3389/fmolb.2020.568777

**Published:** 2020-10-19

**Authors:** James T. Shoemaker, Wanrui Zhang, Selin I. Atlas, Richard A. Bryan, S. Walker Inman, Jelena Vukasinovic

**Affiliations:** ^1^Lena Biosciences, Inc., Atlanta, GA, United States; ^2^Lucid Scientific, Atlanta, GA, United States

**Keywords:** 3D cell culture, organ-on-a-chip, liver model, CYP450, drug metabolism, cell metabolism, primary human hepatocytes, blood substitute

## Abstract

Remarkable advances in three-dimensional (3D) cell cultures and organ-on-a-chip technologies have opened the door to recapitulate complex aspects of human physiology, pathology, and drug responses *in vitro*. The challenges regarding oxygen delivery, throughput, assay multiplexing, and experimental complexity are addressed to ensure that perfused 3D cell culture organ-on-a-chip models become a routine research tool adopted by academic and industrial stakeholders. To move the field forward, we present a throughput-scalable organ-on-a-chip insert system that requires a single tube to operate 48 statistically independent 3D cell culture organ models. Then, we introduce in-well perfusion to circumvent the loss of cell signaling and drug metabolites in otherwise one-way flow of perfusate. Further, to augment the relevancy of 3D cell culture models *in vitro*, we tackle the problem of oxygen transport by blood using, for the first time, a breathable hemoglobin analog to improve delivery of respiratory gases to cells, because *in vivo* approximately 98% of oxygen delivery to cells takes place via reversible binding to hemoglobin. Next, we show that improved oxygenation shifts cellular metabolic pathways toward oxidative phosphorylation that contributes to the maintenance of differentiated liver phenotypes *in vitro*. Lastly, we demonstrate that the activity of cytochrome P450 family of drug metabolizing enzymes is increased and prolonged in primary human hepatocytes cultured in 3D compared to two-dimensional (2D) cell culture gold standard with important ramifications for drug metabolism, drug-drug interactions and pharmacokinetic studies *in vitro*.

## Introduction

Biotransformation of xenobiotics by hepatic metabolism is a critical step in drug disposition. The modification of drug molecules by liver microsomal and non-microsomal enzymes determines its efficacy, toxicity, and pharmacokinetics. Cytochrome P450 (CYP450), a family of heme-containing microsomal enzymes, plays a major role in Phase I biotransformation of xenobiotics by catalyzing numerous types of reactions, such as oxygenation, dehydrogenation, and reduction (Gibson and Skett, 2001; McDonnell and Dang, 2013). Due to their vital role in *human* drug metabolism, poor activity of drug metabolizing enzymes *in vitro* remains a fundamental bottleneck.

The predominant reactions catalyzed by Phase I drug metabolism are oxidative, with the basic reaction mechanism given by Veith and Moorthy (2018):

$$R\,H+2\,H^{+}+O_{2}+2\,e^{-}\to R\,O\,H+H_{2}\,O$$

Thus, oxygen availability is critical for proper enzyme function. Our goal was to augment CYP450 activity *in vitro* by (1) increasing the intracellular oxygen pool, while (2) keeping reactive oxygen species (ROS) within usual levels. We further wanted to achieve this goal by recognizing the need for compliance with standard operating procedures. Specifically, we wanted to enhance oxygen supply to cultures while maintaining the same volume of medium as in standard multi-well plates for which CYP450 benchmarks already exist. To solve this problem, we introduce the PerfusionPal insert that uses the same volume of medium as in a multi-well plate, but positions the cells in the middle of the column of medium, and incorporates Blood Substitute, a hemoglobin analog, below the culture medium. The PerfusionPal oxygen delivery/CYP450 activity solution works on four levels. First, PerfusionPal provides access to oxygen from both the apical and the basal sides of cultures. Second, the distance from the top of the culture to the air interface is halved compared to a multi-well plate, enhancing apical oxygen availability. Third, Blood Substitute addresses limited oxygen solubility in the culture medium and delivers oxygen to the basal side of cultures. Fourth, when perfusion is turned on, pericellular oxygen delivery is enhanced by in-well perfusion.

Understanding the interaction with and the induction of CYP450 enzymes for investigational drugs is the key to drug development and drug-drug interactions according to the guideline of the U.S. Food and Drug Administration (FDA) (FDA, 2020). Despite the considerable efforts in developing *in vitro* CYP450 metabolism assays, faithfully charactering CYP450 induction and Phase I drug metabolism remains a challenge.

Primary human hepatocytes (PHH) are the gold standard for drug metabolism studies. However, hepatocyte viability and their CYP450 expression drop drastically and rapidly, as early as after 4 h in traditional cell culture systems (Rodriguez-Antona et al., 2002). This limits the duration and accuracy of studies, and therefore limits the robustness of PHH as a model for drug metabolism and drug-drug interaction studies *in vitro*. The use of alternative cell-based systems and hepatocyte fractions has numerous shortcomings that outweigh their benefits over PHH models. Hepatoma cell lines have distinct biological regulation of CYP450 induction that is different from that in PHH and *in vivo*. In addition, the majority of cell line models suffer from negligible CYP450 activities compared to PHH under baseline and induced conditions (Rodriguez-Antona et al., 2002; Gerets et al., 2012; Kvist et al., 2018). The only exceptions are HepaRG cells, a hepatic stem cell line, that was, with certain limitations, characterized as a viable alternative (Berger et al., 2016), and expandable human hepatocytes (Levy et al., 2015; Zhang et al., 2018). The use of induced pluripotent stem cells (iPSCs) differentiated into hepatocyte-like-cells is also gaining popularity. iPSC-derived hepatocytes express many characteristics of PHH, such as albumin secretion and lipid storage, and overcome PHH limitations in the context of cell expansion (Shan et al., 2013). However, iPSC-derived hepatocytes more closely resemble the immature phenotype rather than adult hepatocytes (Shan et al., 2013). Their CYP450 activity, while higher than that of immortalized cell lines, is still greatly reduced compared to PHH. Hepatocyte fractions, purified human liver microsomes, lack cytosolic enzymes and several cofactors required for drug metabolism (Ohkura et al., 2014). They can resolve transient metabolic responses that last from only minutes up to 1 h (Yadav et al., 2020). This time period is too brief to gain a mechanistic understanding of the processes in question, leaving cellular systems as a necessity for mechanistic research. Maintaining differentiated phenotypes of PHH *in vitro* is, therefore, vital for studies of *human* drug metabolism, drug and metabolite toxicity, and drug–drug interactions because animals have different isoforms of drug metabolizing enzymes, clearance, and metabolites (Martignoni et al., 2006; Li, 2008). Collectively, there is an urgent need to engineer advanced, physiologically more representative *in vitro* PHH systems to resolve the challenge of declining cell functions in order to advance our understanding of human drug metabolism and chronic liver diseases.

In 2D cell culture, PHHs have the highest CYP450 activities within hours after thawing or isolation in cryopreserved and freshly isolated cells, respectively. In suspension hepatocytes, the activity begins to decay 6 h after recovery on day 0 (Kidambi et al., 2009), and is negligible by day 1. In plateable hepatocytes, CYP450 activity lasts longer than in suspended hepatocytes, but by day 1, the activity is generally below 50% of day 0 values, and decays rapidly thereafter. To date, numerous approaches have been used to augment and extend PHH function *in vitro*. Collagen-Matrigel^®^ overlay, a hepatocyte “sandwich culture system,” has been used for long-term maintenance of PHH. The system mimics the extracellular matrix found *in vivo*, lining the space of Disse, which directly interacts with hepatocytes (Bale et al., 2016). Other approaches to long term culture of hepatocytes are three-dimensional (3D) cell culture systems, including: liver spheroids, 3D scaffolds, co-cultures, bioreactors, and organ-on-a-chip type devices (Beckwitt et al., 2018; Ehrlich et al., 2019). While each of these systems have their advantages and shortcomings, considering experimental complexity and the needs for reasonable throughput for drug metabolism studies, liver spheroids co-cultured with feeder cells have had perhaps the most remarkable drug metabolism results (Ohkura et al., 2014; Berger et al., 2016). The advantages of using spheroids are that they possess cell-cell interactions and can demonstrate liver regeneration and development (Deng et al., 2019). Disadvantages to 3D spheroids are that the cells aggregate arbitrarily, and uniform spheroid generation is difficult. Additionally, cells become necrotic in the center of large aggregates. It was shown that aggregates with diameters lager than 150 μm typically possess a necrotic core due to poor diffusion of oxygen and nutrients (Tasnim et al., 2020).

Oxygen availability is critical for cell survival and function *in vivo* and *in vitro* (Place et al., 2017). This is especially true for primary human cells that are highly dependent on oxidative phosphorylation (OXPHOS) for energy production. For this reason, PHH have an oxygen consumption rate (OCR) that is 12 times higher than the commonly used hepatoma cell line HepG2/C3A, and almost 5 times higher than HepaRG cells (Ehrlich et al., 2019). Microfluidic and organ-on-a-chip devices attempt to meet this cellular need by introducing perfusion, a forced convection flow of medium to enhance delivery of dissolved oxygen to cells (Beckwitt et al., 2018). While helpful, this is not an effective way of delivering oxygen to cells in culture. *In vivo*, dissolved oxygen in blood meets only about 2% of total cellular demands for oxygen (Pittman, 2011). Therefore, a microfluidic device needs to employ a prohibitively high flow rate of medium to enhance oxygen solubility and provide a sufficient amount of oxygen to cells. This is far from physiological conditions. High pressure and flow rate lead to extensive dilution of metabolites (Ehrlich et al., 2019) and to cellular injury due to high normal and shear stresses (Rambani et al., 2009; Ehrlich et al., 2019). *In vivo*, about 98% of the oxygen supply to cells and tissues comes from the oxygen reversibly bound to hemoglobin (Pittman, 2011). Hence, the use synthetic oxygen carriers is a more effective method of oxygen delivery to cultured cells. This approach has been used successfully in bioartificial liver bioreactors to meet hepatocyte oxygen demands and preserve differentiated phenotypes (Nieuwoudt et al., 2005; Kinasiewicz et al., 2008; Nieuwoudt et al., 2009). Unfortunately, common oxygen carriers *in vivo* and *in vitro* are emulsified perfluorocarbons (PFC) that, as emulsions, require excessive equipment in order to charge them with oxygen (Shi and Coger, 2013), and are thus not readily amenable to multi-well plate based cell culture and drug testing methodologies.

Here, we present a novel solution that solves the opposing requirements of hemoglobin-based oxygen delivery and cell function versus experimental complexity and routine culture workflow. Lena Biosciences’ proprietary and patented (U.S. Patent No. 9,701,938) organ-on-a-chip insert system, PerfusionPal, uses a unique in-well perfusion method that concentrates drug metabolites and provides both dissolved oxygen and a hemoglobin-like oxygen supply to hepatocytes analogous to the *in vivo* environment.

**FIGURE 1:**
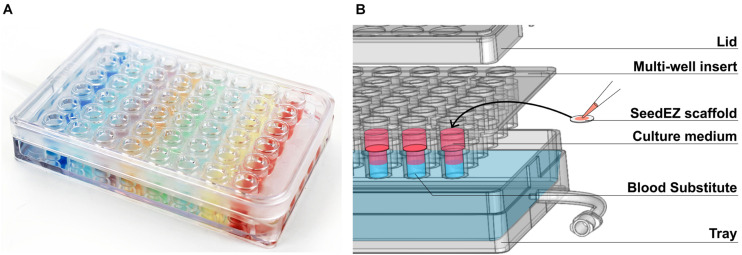
The PerfusionPal insert system. **(A)** Photo of the 48-well system illustrating the absence of crosstalk between wells using colored indicators. **(B)** Exploded diagram of the 48-well system showing the three components (Lid, Multi-well insert, and Tray) as well as the SeedEZ scaffold and an illustration of the culture medium floating atop the dense, immiscible, and insoluble Blood Substitute.

**FIGURE 2:**
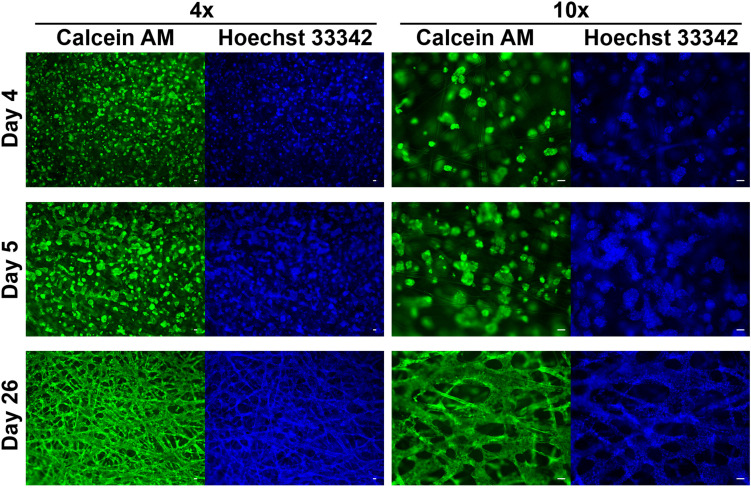
HepG2 cells grown in SeedEZ scaffold labeled with Calcein AM and Hoechst 33342 nuclear stain. Three time points post-plating are indicated as well as magnification. Cells begin forming spheroids soon after plating, begin to merge, and ultimately form a dense tissue-like construct. Scale bars: 50 μm.

**FIGURE 3:**
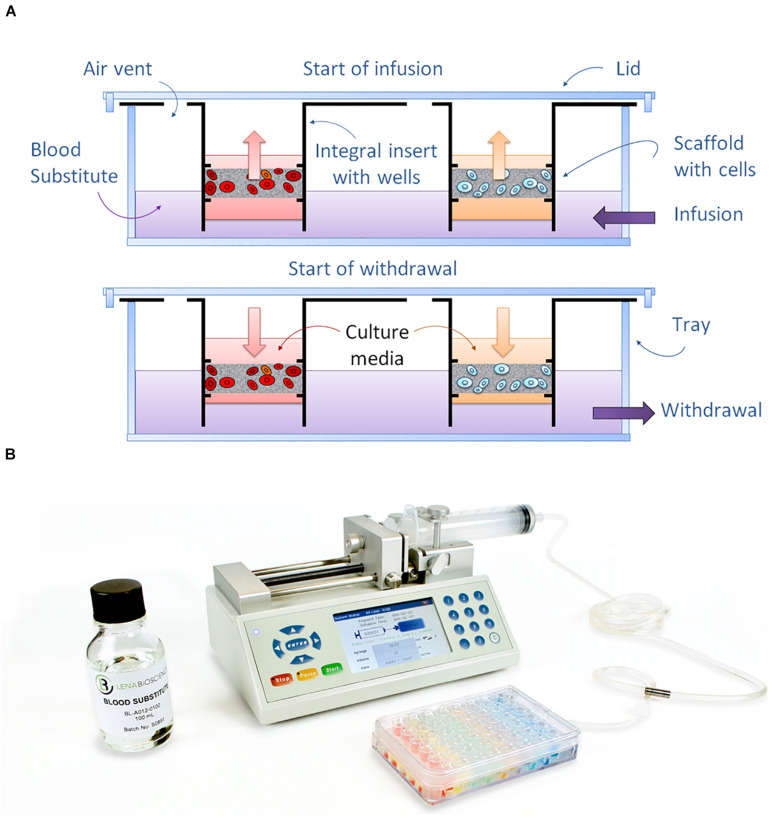
Principle of operation of the PerfusionPal system. **(A)** Diagram depicting how the dense Blood Substitute acts as a piston to drive the flow of medium up and down within each independent well using just a single tube and pump. **(B)** Perfused Organ Panel starter configuration showing the PerfusionPal insert system, Blood Substitute, and bi-directional syringe pump.

The PerfusionPal insert system (Figure 1A) comprises an integral multi-well insert, a tray, and a lid (Figure 1B). Each well has Lena Biosciences’ 3D cell culture scaffold, SeedEZ (U.S. Patent No. 9,334,473), that is fixed in place at approximately mid-height of the well. SeedEZ is a 500 μm thick, transparent, hydrophilic, glass micro-fiber scaffold in which cells can be seeded in medium or hydrogel to encourage both cadherin-mediated cell-cell and integrin-mediated cell-extracellular matrix (ECM) signaling (Didier et al., 2020). SeedEZ is optimized for long-term growth of 3D cell cultures and complex 3D co-culture models of brain, cancer, liver, bone, connective tissues, etc. (Roberts et al., 2018; Shoemaker et al., 2018; Vukasinovic et al., 2018; Lang et al., 2019; Xiong et al., 2019; Didier et al., 2020; Jensen and Teng, 2020), and as shown herein for weeks-long growth of HepG2 cells, a hepatocellular carcinoma liver cell line (Figure 2). SeedEZ scaffolds are easy to handle. Analogous to a piece of paper, the scaffolds can be easily transferred dish-to-dish using sterile forceps without damaging the cultures, and taken out of the PerfusionPal insert and transferred to another dish for cell isolation and high-resolution imaging. Compared to spheroid models, SeedEZ is not limited by cell types that aggregate. Compared to hydrogels, SeedEZ provides consistent 3D cell cultures with defined dimensions and reproducible cell distribution in x, y, and z. Cultures in SeedEZ are also protected because they cannot be aspirated into a pipet tip during media changes as spheroids can, and they cannot be peeled off the dish as hydrogels can in extended culture. Cells seeded in the scaffold are maintained in statistically independent wells in their own medium that floats atop a high-density Blood Substitute PFC that is twice as dense as water and in which medium, reagents, and drugs are immiscible and insoluble. Cyclic infusion and withdrawal of the Blood Substitute into and from the tray introduces cyclic up and down in-well perfusion into a multi-well insert (Figure 3A). The principle of operation is simple: raising the PFC level in the tray raises the media levels in all insert wells; lowering the PFC level in the tray lowers the media levels in all insert wells. PFC is cyclically pushed in and pulled out of the tray using a push/pull syringe pump (Figure 3B). The lowest level of media in the wells is at the start of the PFC infusion stroke, and the highest level of media in the wells is at the start of the PFC withdrawal stroke (Figure 3A). During perfusion, the flow rates are calculated to produce a 2 mm upward or downward medium displacement, corresponding to 16 culture volume changes per day. All cultures are perfused in their own medium, and the PFC does not contact the cultures at any time during perfusion.

Since the wells are statistically independent, and the only liquid infused and withdrawn from the tray is the Blood Substitute, PerfusionPal requires just one pump and one tube to operate 48 statistically independent cultures as shown with our Perfused Organ Panel starter configuration (Figure 3B). Without the need for separate tubes and pumps for 48 culture/well conditions, PerfusionPal is simple to set up and use. All culturing, media changes, and assaying is done as in any multi-well plate, and SeedEZ scaffolds are portable and removable from the insert for high-resolution imaging. Unlike organ-on-a-chip devices with unidirectional flow, PerfusionPal does not wash away signaling molecules secreted by the cells, maintaining critically important cell-cell communication pathways, and concentrating the secretome and metabolome for improved cell signaling, chemical and drug metabolism studies *in vitro*. Since the Blood Substitute does not support the growth of microorganisms, the risk of infection is negligible compared to all perfusion systems that use medium in tubes, syringes, and moving pump components. Lastly, since there are no micro-components or micro-channels to trap gas or clog due to non-specific protein adsorption to the interior of small channels, PerfusionPal has consistent and reliable long-term operation.

The fact that PerfusionPal requires only one tube and one pump to simultaneously perfuse all cultures in their own media is because the Blood Substitute acts as a piston in each culture well. Although there is no mixing of the liquid phase, the Blood Substitute dissolves orders of magnitude more air than culture medium. O_2_ solubility is 78 times higher than in water (370 ml/L vs. 4.74 ml/L) (Heintz, 2012; FLUOROMED, 2020). CO_2_ solubility is slightly higher than that of O_2_ (Heintz, 2012), allowing for superior pH maintenance in bicarbonate buffer systems. The Blood Substitute is a completely inert, non-polar, synthetic PFC compound, perfluoroperhydrophenanthrene, that is both hydrophobic and lipophobic (Riess, 2005; Spiess, 2009). The only molecules that are soluble in Blood Substitute at very low levels are aliphatic hydrocarbons and heavily halogenated organic compounds, but these molecules are not commonly used in cell culture. Chloroform used for nucleic acid extraction is an exception. Therefore, SeedEZ scaffolds with 3D cell cultures need to be removed from the PerfusionPal insert and transferred to a tube prior to RNA extractions using chloroform.

In cell culture, Blood Substitute acts as hemoglobin, but with the ability to passively accept *all* respiratory gases because it is constantly equilibrated with air in our ventilated, open system perfusion platform that eliminates the need for large scale equipment to charge PFC emulsions with 95% oxygen. The Blood Substitute is similar to gas-carrier PFC liquids used in neonatal liquid ventilation (Hirschl et al., 1995; Shaffer et al., 1999; Hirschl, 2017; Eichenwald et al., 2020; Kohlhauer et al., 2020), liquid breathing (Kylstra, 1974), lung atelectasis (Henrichsen et al., 2012; Bali et al., 2017), organ preservation (Matsumoto and Kuroda, 2002; Matsumoto, 2005), and artificial blood (Riess, 2005; Spiess, 2009, 2010), but contains only one molecule with two elements, carbon and fluorine. When used in clinical or *in vivo* research applications, PFCs do not mix with any bodily fluids or enter tissues, and do not support microbial growth. They are passively expelled by exhalation or by transpiration through the skin (Biro and Blais, 1987; Lowe, 2003; Li et al., 2007; Kazzaz et al., 2011). Lastly, the specific molecule used in Blood Substitute exhibits a negligible amount of evaporation due to its near-zero vapor pressure at 37°C, allowing for long-term culture studies without replenishment.

## Materials and Methods

### Cell Culture

All maintenance and experimental culturing was carried out in a humidified, 37°C, 5% CO_2_ incubator (VWR, Radnor, PA, United States). HepG2 cells (ATCC^®^ HB-8065^TM^, Manassas, VA, United States) were maintained in EMEM (Corning Life Sciences, Tewksbury, MA, United States) supplemented with heat-inactivated 10% fetal bovine serum (FBS, HyClone^TM^, GE Healthcare, Chicago, IL, United States). Cryoplateable PHH (Liverpool^TM^ 10-donor pool, mixed gender, BioIVT, Hicksville, NY or *In Vitro* ADMET Laboratories, Columbia, MD, United States) were cultured in InVitroGRO CP medium (BioIVT) or Hepatocyte Induction Medium (HIM^TM^, *In Vitro* ADMET Laboratories). BT-474 cells (ATCC^©^ HTB-20^TM^) were maintained in DMEM/F12 (Corning Life Sciences) supplemented with 10% FBS and 2 mM GlutaMAX (Gibco, ThermoFisher, Waltham, MA, United States). NoSpin HepaRG^TM^ cells (TRL/Lonza, Basel, Switzerland) and iCell^®^ Hepatocytes 2.0 (FUJIFILM Cellular Dynamics, Inc., Madison, WI, United States), were cultured in the manufacturer’s recommended media.

For imaging studies (Figure 2), 500,000 HepG2 cells were plated in 3.75 mg/mL Matrigel^®^ (Corning Discovery Labware, Bedford, MA, United States) in 9.5 mm SeedEZ scaffolds and cultured for 4–26 days before imaging.

**FIGURE 4:**
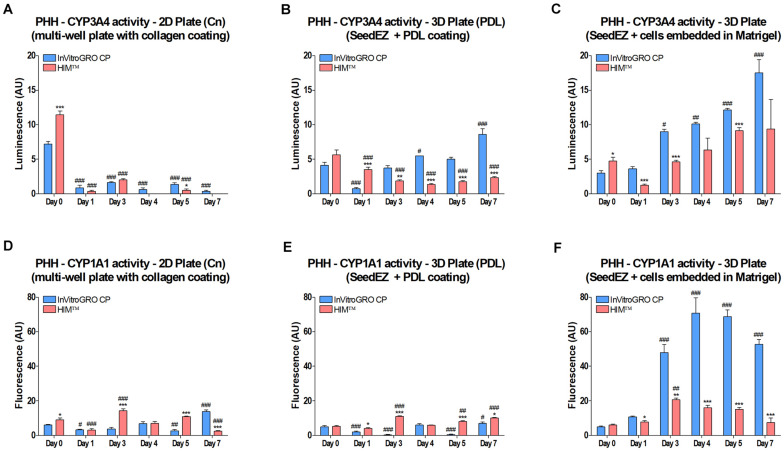
Effect of 3D culturing on CYP450 activity in PHH. Cells were **(A,D)** plated into a multi-well plate with a collagen coating, **(B,E)** seeded into PDL-coated SeedEZ scaffolds, or **(C,F)** suspended in Matrigel^®^ and delivered into SeedEZ scaffolds, and grown in either serum-containing (InVitroGRO CP) or serum-free (HIM^TM^) medium. **(A-C)** CYP3A4 and **(D-F)** CYP1A1 activity were measured at the indicated time points for cells plated as shown. *p < 0.05, **p < 0.01, ***p < 0.001 comparing the effect of the different media on CYP450 activity at each time point; ^#^*p* < 0.05, ^##^*p* < 0.01, ^###^*p* < 0.001 comparing CYP450 activity at each time point to day 0 for the same plating condition and medium.

For baseline CYP450 activity studies (Figure 4), 100,000 PHH were seeded into collagen-coated 48-well plates (*In Vitro* ADMET Laboratories), seeded in 8 mm SeedEZ scaffolds coated with poly-D-lysine (MW > 300,000, 100 μg/mL, Millipore Sigma, Burlington, MA, United States) and the scaffolds placed into 48-well plates, or suspended in 8 mg/mL Matrigel^®^ that was delivered into 8 mm SeedEZ scaffolds and the scaffolds placed into 48-well plates. Cells were cultured in serum-containing InVitroGRO CP medium or in serum-free HIM^TM^ and assayed for CYP3A4 and CYP1A1 activity as described below.

**FIGURE 5:**
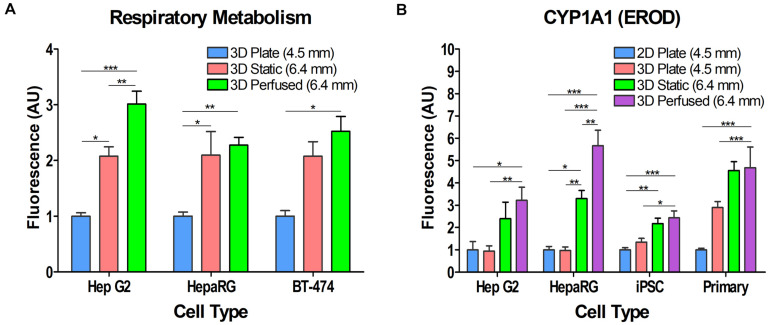
Effect of Blood Substitute and perfusion on respiratory metabolism and CYP1A1 activity in various cell types. **(A)** Respiratory metabolism as measured by alamarBlue^®^ is increased in HepG2, HepaRG, and BT-474 cells when cultured with Blood Substitute and perfusion. **(B)** CYP1A1 activity is increased in HepG2, HepaRG, iPSC-derived hepatocytes, and PHH when cultured with Blood Substitute and perfusion. The column of medium in the 12-well PerfusionPal insert (3D Static and 3D Perfused) is 1.4 times larger than in the 24-well plate (2D Plate and 3D Plate). The number in parentheses indicates the medium column height for each condition. *p < 0.05, **p < 0.01, ***p < 0.001.

For respiratory metabolism measurements (Figure 5A), 333,000 cells were suspended in 8 mg/mL Matrigel^®^ and delivered into 12.7 mm SeedEZ scaffolds and cultured in a 24-well plate (3D Plate) or in a 12-well PerfusionPal insert without (3D Static) or with (3D Perfused) perfusion for 5 (HepaRG), 7 (BT-474), or 10 (HepG2) days. An alamarBlue^®^ assay was performed as described below. For CYP1A1 activity measurements (Figure 5B), 333,000 cells were plated in 2D in a 24-well plate (2D Plate) or suspended in 8 mg/mL Matrigel^®^ and delivered into 12.7 mm SeedEZ scaffolds that were placed in a 24-well plate (3D Plate) or placed in a 12-well PerfusionPal insert without (3D Static) or with (3D Perfused) perfusion for 5 (HepaRG, iPSC, and PHH) or 7 (HepG2) days. CYP1A1 activity was determined as described below. For both studies, the medium volume was the same for all conditions. However, in the absence of an adequate multi-well plate control having an identical well diameter as that of the PerfusionPal insert well, the total medium column height, including the scaffold thickness, in the 12-well PerfusionPal insert (3D Static and 3D Perfused) was 1.4 times larger than that in the 24-well plate used for 2D Plate and 3D Plate controls.

**FIGURE 6:**
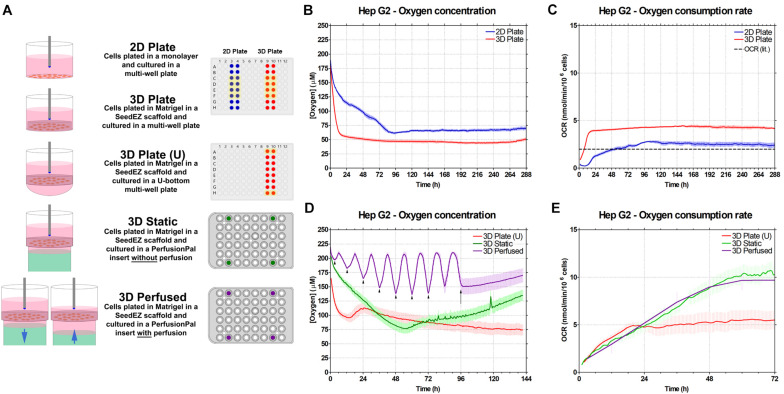
Effect of Blood Substitute and perfusion on oxygen availability and oxygen consumption rate in HepG2 cells. **(A)** Graphic representation of the experimental setup for experiments comparing 2D and 3D culture (2D Plate vs. 3D Plate) and comparing the additions of Blood Substitute and perfusion to 3D culture (3D Plate (U) vs. 3D Static vs. 3D Perfused). The position of the oxygen sensing probe is depicted in each well. Color-filled wells in the multi-well plate and the PerfusionPal insert diagrams indicate the positions of the probes. Wells highlighted in yellow were included in the data plots and analysis. **(B)** Oxygen concentration measurements for 2D Plate and 3D Plate conditions. **(C)** OCR for 2D Plate and 3D Plate conditions. The literature value of OCR for HepG2 cells is indicated by the dashed line. **(D)** Oxygen concentration measurements for 3D Plate (U) vs. 3D Static vs. 3D Perfused conditions. The 3D Perfused condition shows a sinusoidal pattern matching the period of perfusion. The minima and maxima correspond to the ALI being the furthest and the closest to cultures, respectively. When the ALI is the furthest from cultures, the configuration matches that of the 3D Static condition. The OCR value for these points (arrowheads) were plotted for the 3D Perfused condition. Perfusion was stopped at 96 h (arrow), after which the oxygen concentration began to steadily rise due to evaporation. **(E)** OCR for 3D Plate (U) vs. 3D Static vs. 3D Perfused conditions.

For dissolved oxygen (DO) measurements comparing 2D and 3D culture (Figure 6B), 56,250 HepG2 cells were plated in 2D in a 96-well plate (2D Plate) or suspended in 8 mg/mL Matrigel^®^, delivered into 6 mm diameter SeedEZ scaffolds (3D Plate), and cultured in a 96-well plate for 12 days. For DO measurements assessing the effect of adding Blood Substitute and perfusion to 3D cell cultures (Figure 6D), 100,000 HepG2 cells were suspended in 8 mg/mL Matrigel^®^, delivered into 8 mm diameter SeedEZ scaffolds, and cultured in a 96-well U-bottom plate (8 mm well diameter) [3D Plate (U)] or in a 48-well PerfusionPal insert (8 mm well diameter) without (3D Static) or with (3D Perfused) perfusion for 6 days. DO concentration was measured as described below.

**FIGURE 7:**
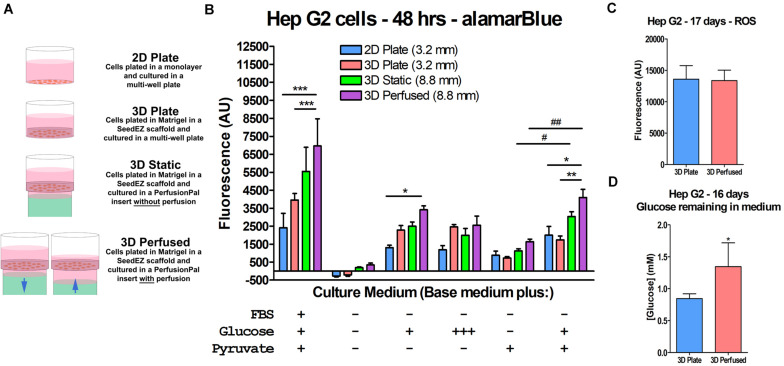
Increased oxygen availability and perfusion influence HepG2 cell metabolism. **(A)** Graphic representation of the culture conditions used in the cell metabolism study and in other studies described later. **(B)** Cell metabolism data illustrating the effect of Blood Substitute and perfusion on metabolism as measured by alamarBlue^®^. Media conditions are indicated for each group of culture conditions. The conditions were: EMEM with FBS, low glucose, and pyruvate; unsupplemented DMEM; DMEM with low glucose; DMEM with high glucose; DMEM with pyruvate; and DMEM with low glucose and pyruvate. The column of medium in the 48-well PerfusionPal insert (3D Static and 3D Perfused) was 2.8 times larger than in the 48-well plate (2D Plate and 3D Plate). The number in parentheses indicates the medium column height for each condition. **(C)** ROS measurements from a long-term culture experiment comparing 3D Plate to 3D Perfused conditions, indicating that despite the increased oxygen availability, ROS production does not change. **(D)** Glucose measurements from the same study showing that glucose consumption is lower in the 3D Perfused condition. *p < 0.05, **p < 0.01, ***p < 0.001 for 3D Perfused conditions compared to other culture conditions within each medium condition; ^#^p < 0.05, ^##^p < 0.01 for 3D Static and 3D Perfused conditions culture with pyruvate and with and without low glucose.

For metabolism studies (Figure 7B), 100,000 HepG2 cells were plated in 2D in a 48-well plate (2D Plate) or suspended in 8 mg/mL Matrigel^®^, delivered into 8 mm SeedEZ scaffolds, and cultured in a 48-well plate (3D Plate) or in a 48-well PerfusionPal insert without (3D Static) or with (3D Perfused) perfusion for 48 h in maintenance medium or in glucose-free and pyruvate-free DMEM without supplement or supplemented with 5.5 mM glucose, 25 mM glucose, 5.5 mM sodium pyruvate, or a combination of both. Matrigel^®^ volume was replaced with HBSS for 2D conditions. Matrigel^®^ stock was diluted to 16 mg/ml in HBSS for 3D conditions (8 mg/mL final concentration with cells). The medium volume was the same for all conditions, but without an adequate multi-well plate control having the same well diameter as that of PerfusionPal, the 48-well PerfusionPal insert (3D Static and 3D Perfused) had a 2.8 times larger medium column than the 48-well plate used for 2D Plate and 3D Plate controls. An alamarBlue^®^ assay was run after 48 h as described below. For ROS and glucose studies (Figures 7C,D), 333,000 HepG2 cells were suspended in 8 mg/mL Matrigel^®^ and delivered into 12.7 mm diameter SeedEZ scaffolds and cultured in a 24-well plate or in a 12-well PerfusionPal insert with perfusion for 17 days.

**FIGURE 8:**
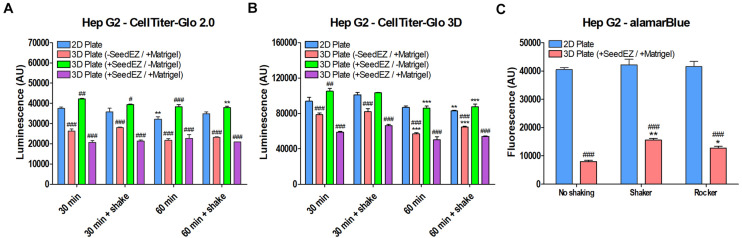
Comparison of multiple assays in 2D and 3D demonstrates signal reduction in 3D due to the presence of Matrigel^®^. HepG2 cells plated in 2D, in Matrigel^®^, in SeedEZ, or in SeedEZ with Matrigel^®^ were assayed using the lytic assays: **(A)** CellTiter-Glo 2.0 and **(B)** CellTiter-Glo 3D for the indicated times with and without shaking. *p < 0.05, **p < 0.01, ***p < 0.001 comparing different reaction conditions (time and agitation) for the same culture method; ^#^*p* < 0.05, ^##^*p* < 0.01, ^###^*p* < 0.001 comparing different culture methods for the same reaction conditions. **(C)** AlamarBlue^®^ cell metabolism assay in HepG2 cells plated in 2D or in a SeedEZ scaffold with Matrigel^®^ without agitation or with shaking or rocking. *p < 0.05, **p < 0.01 for shaker and rocker conditions compared with the condition without agitation. ^###^*p* < 0.001 for comparing 3D with 2D for each agitation condition.

For 2D vs. 3D assay characterizations (Figure 8), 100,000 HepG2 cells were plated in 2D in a 48-well plate, suspended in 8 mg/mL Matrigel^®^ and plated in a 48-well plate or seeded in an 8 mm SeedEZ scaffold, or suspended in 8 mg/mL Matrigel^®^ and delivered into an 8 mm SeedEZ scaffold in a 48-well plate. After 1 h, CellTiter-Glo^®^ or alamarBlue^®^ assays were run as described below.

**FIGURE 9:**
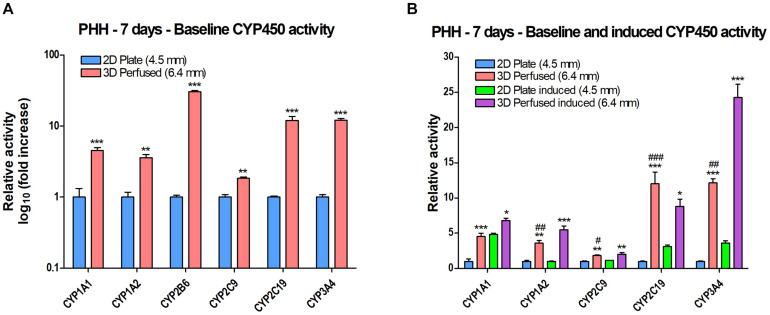
CYP450 activity of PHH grown in 2D in a 24-well plate (2D Plate) or in 3D in a 12-well PerfusionPal insert (3D Perfused) after 7 days. The column of medium in the 3D Perfused condition is 1.4 times larger than in the 2D Plate condition. The number in parentheses indicates the medium column height for each condition. The assays in 3D Perfused cultures were run as in 2D Plate cultures after the transfer of SeedEZ scaffolds into a 24-well plate. **(A)** Baseline (uninduced) activity showing significant increases in PerfusionPal for each CYP450 enzyme tested. **p < 0.01, ***p < 0.001 comparing 2D Plate to 3D Perfused condition. **(B)** Perfusion augments baseline and induced CYP450 activity. *p < 0.05, **p < 0.01, ***p < 0.001 comparing 3D Perfused conditions (uninduced and induced) to their respective 2D Plate controls. ^#^*p* < 0.05, ^##^*p* < 0.01, ^###^*p* < 0.001 comparing 3D Perfused uninduced with 2D Plate induced for each CYP450 enzyme.

**FIGURE 10:**
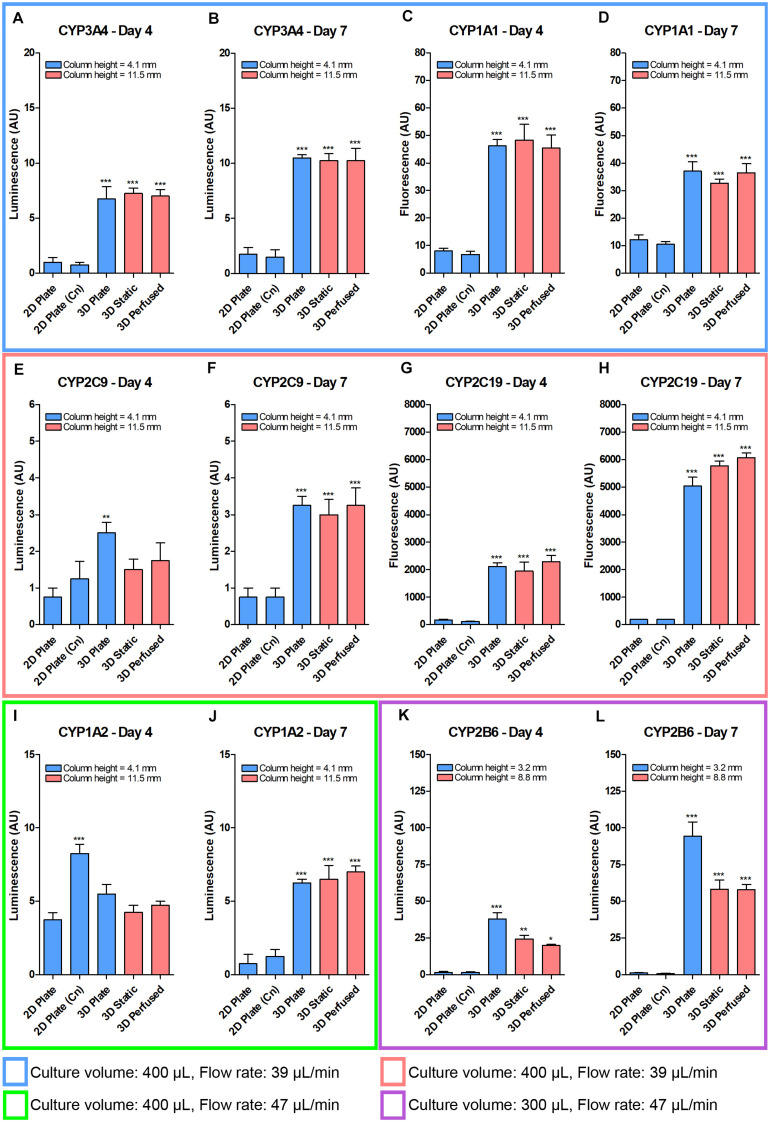
Despite a 2.8 times larger column of medium, the baseline CYP450 PHH activity after 4 and 7 days did not drastically decrease in the 48-well PerfusionPal (3D Static and 3D Perfused) compared to 48-well 3D Plate controls. PHH were grown in 2D (without and with collagen (Cn) coating), in 3D in a multi-well plate, and in 3D in the PerfusionPal insert without and with perfusion. The medium column height for each condition is defined in the legend of each graph. Colored boxes indicate different media volumes and flow rates during culturing. 3D Static and 3D Perfused cultures were assayed as in 2D Plate and 3D Plate controls by transferring SeedEZ scaffolds into a 48-well plate. **(A,B)** CYP3A4 and **(C,D)** CYP1A1 assays were multiplexed for cells grown in 400 μL of medium with a flow rate of 39 μL/min. **(E,F)** CYP2C9 and **(G,H)** CYP2C19 assays were multiplexed for cells grown as in **(A–D)**. **(I,J)** CYP1A2 was assayed alone for cells grown as in **(E–H)**, but with a flow rate of 47 μL/min. **(K,L)** CYP2B6 was assayed alone for cells grown as in **(I,J)**, but with a medium volume of 300 μL. *p < 0.05, **p < 0.01, ***p < 0.001 comparing 2D (Cn) and 3D conditions to their respective 2D Plate controls.

**FIGURE 11:**
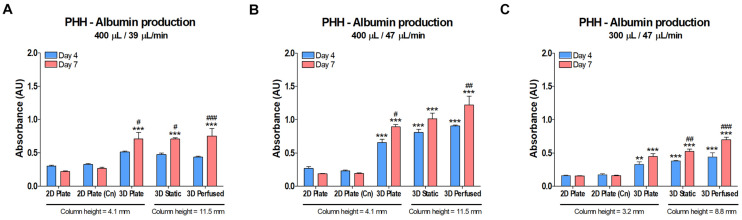
Albumin production in PHH did not decrease in PerfusionPal (3D Static and 3D Perfused) despite a 2.8 times larger medium column than in multi-well plate controls (2D Plate and 3D Plate). The column height for each condition is defined below the graphs. Graphs cannot be compared to each other due to issues with calibration curves (measurements exceeded the calibration range). Prior to performing the CYP450 activity assays on cultures on days 4 and 7 shown in Figure 10, media samples were taken and assayed by ELISA for albumin. **(A)** PHH cultured in 400 μL of medium at a flow rate of 39 μL/min. **(B)** PHH cultured as in **(A)**, but with a flow rate of 47 μL/min. **(C)** PHH cultured as in **(B)**, but with a medium volume of 300 μL. **p < 0.01, ***p < 0.001 comparing 2D Plate (Cn) and 3D conditions to 2D Plate controls. ^#^*p* < 0.05, ^##^*p* < 0.01, ^###^*p* < 0.001 comparing day 4 and day 7 data for each culture condition.

For CYP450 activity and albumin studies, 333,000 (12-well PerfusionPal, Figure 9) or 100,000 (48-well PerfusionPal, Figures 10, 11) PHH were plated in 2D in a 24-well or 48-well plate (2D Plate) or suspended in 8 mg/mL Matrigel^®^, delivered into 12.7 or 8 mm SeedEZ scaffolds, and cultured in a 24-well or 48-well plate (3D Plate) or in a 12-well or 48-well PerfusionPal insert without (3D Static) or with (3D Perfused) perfusion for up to 7 days. 3D cultures were allowed to gel at 37°C for 7–10 min. Matrigel^®^ volume was replaced with HBSS for 2D conditions. Matrigel^®^ stock was diluted to 16 mg/ml in HBSS for 3D conditions (8 mg/mL final concentration with cells). Collagen-coated plates (*In Vitro* ADMET Laboratories) were also used in CYP activity studies in the 48-well format [2D Plate (Cn)].

### Cell Imaging

HepG2 cultures were rinsed twice with DPBS, incubated with dye solution comprising 5 μM Calcein AM green (Corning Discovery Labware) and 10 μM Hoechst 33342 (cell-permeable nuclear stain, Thermo Scientific, Waltham, MA, United States) for 30 min at 37°C, and rinsed twice with DPBS. Imaging was carried out using a Nikon Eclipse 80i upright microscope (Nikon Instruments Inc., Melville, NY, United States) equipped with a MicroFIRE camera (Optronics Inc. Goleta, CA, United States).

### Perfused Organ Panel

PerfusionPal was primed with 50 mL (12-well) or 75 mL (48-well) Blood Substitute. SeedEZ scaffolds seeded with cells in Matrigel^®^ were placed into the wells of a PerfusionPal insert containing 750 μL (12-well) or 300–400 μL (48-well) culture medium. 3D Static conditions were cultured without the perfusion pump. 3D Perfused conditions were cultured with active pumping of Blood Substitute at a target volume and flow rate of 10 mL and 28 μL/min (12-well PerfusionPal) or 14–17 mL and 39–47 μL/min (48-well PerfusionPal), corresponding to two complete perfusion strokes (16 culture volume changes) in a 24-h period.

### Dissolved Oxygen Measurements

Lucid’s real-time oxygen monitoring probes were connected to a handheld device via fiber optic cables (Lucid Scientific, Atlanta, GA, United States). The handheld device powers and transmits light sources from light-emitting diodes (LEDs) onto the probe tips, where the oxygen sensing materials are located. Upon reacting with oxygen, the sensing materials emit, and the fluorescence radiation is transmitted back via the fiber optic cables to the device. The signal is processed and analyzed in real-time by Lucid’s software to provide oxygen concentration throughout the entire experiment. Measurements were taken in one well per second. OCR was calculated from oxygen flux which was determined using oxygen concentration measurements over time and the movement of the oxygen probes (amplitude: 400 μm, period: 30 min). Data were re-sampled at 30 min intervals for analysis.

### AlamarBlue^®^ Assay for Metabolic Function

AlamarBlue^®^ reagent (Bio-Rad, Hercules, CA, United States) was added to the cells at a volume equal to 1/10^th^ of the culture medium volume. The cultures were returned to the incubator for a period of 1–2 h, depending upon cell type. A sample of the medium from each culture was transferred to a black-wall 96-well plate and fluorescence (545 nm excitation/590 nm emission) was read on a BioTek Synergy 4 plate reader (Winooski, VT, United States).

### ROS Assay

H_2_DCFDA was used to measure ROS production. Upon exposure to ROS, the chemical is converted to the highly fluorescent DCF molecule. After washing with HBSS, HepG2 cells cultured in SeedEZ scaffolds in PerfusionPal or in a multi-well plate were incubated with 5 mM of H_2_DCFDA (Invitrogen, Carlsbad, CA, United States) at room temperature for 20 min. The scaffolds were transferred to a black-wall 24-well plate and the fluorescence (485 nm excitation/530 nm emission) was read on a BioTek Synergy 4 plate reader.

### Glucose Assay

The concentration of glucose in medium was assayed using a Glucose-Glo^TM^ assay (Promega, Madison, WI, United States) according to the manufacturer’s instructions.

### CellTiter-Glo^®^ Assay

CellTiter-Glo^®^ 2.0 and CellTiter-Glo^®^ 3D assays (Promega) were performed 1 h after cell seeding according to the manufacturer’s instructions. Assays were run for 30 or 60 min, with or without shaking.

### CYP Activity Measurements

CYP1A1 and CYP2C19 activities were measured fluorescently using ethoxyresorufin-*O*-deethylase (EROD) and 3-cyano-7-ethoxycoumarin (CEC) assays, respectively. For the EROD assay, cells were exposed to 7-ethoxyresorufin (10 μM, AnaSpec, Fremont, CA, United States) and salicylamide (inhibitor of phase II metabolism of resorufin, 1.5 mM, VWR) for 4 h. For the CEC assay, cells were exposed to CEC (25 μM) for 4 h. CYP1A2, CYP2B6, CYP2C9, and CYP3A4 activities were measured using CYP P450-Glo^TM^ assays (Promega) according to the manufacturer’s instructions. Omeprazole (20 μM) was used to induce CYP1A2. Phenobarbital (1 mM) was used to induce CYP2B6. Rifampin (10 μM) was used to induce all other CYP enzymes. Fluorescent and luminescent reads were performed on a BioTek Synergy 4 plate reader.

### Albumin ELISA

The supernatants were collected after the PHH were cultured for 4 and 7 days. The human albumin concentration in the supernatants was determined using the Human Serum Albumin DuoSet ELISA Kit (R&D Systems, Minneapolis, MN, United States) according to the manufacturer’s instructions. The assay was confirmed to be human-specific as there was no signal with FBS-containing blanks. Samples were analyzed without dilution for each condition.

### Statistical Analysis

Statistical significance was determined by Student’s *t*-test, One-way ANOVA and Tukey’s *post hoc* test, or Two-way ANOVA and Bonferroni’s post-test test for multiple comparisons.

## Results

For all studies, the tests and controls are defined in Table 1. The abbreviations used to characterize experimental geometry are defined in Table 2.

**TABLE 1:** Test and control definitions.

**Controls**	
2D Plate	The cells were plated into tissue cultured treated (TCT) multi-well plates.
2D Plate (Cn)	The cells were plated into collagen-coated plates.
3D Plate	The cells were suspended in 8 mg/ml Matrigel^®^ and delivered into SeedEZ scaffolds that were placed into TCT multi-well plates.
3D Plate (PDL)	The cells were delivered into Poly-D-Lysine (PDL) coated SeedEZ scaffolds that were placed into TCT multi-well plates.
3D Plate (U)	The cells were suspended in 8 mg/ml Matrigel^®^ and delivered into SeedEZ scaffolds that were placed into U-bottom plates such that the culture medium was above and below the scaffold.
**Tests**	
3D Static	The cells were suspended in 8 mg/ml Matrigel^®^ and delivered into SeedEZ scaffolds that were placed into PerfusionPal insert such that the culture medium was above and below the scaffold. The Blood Substitute was positioned underneath the culture medium, but the cultures were not perfused.
3D Perfused	The cells were suspended in 8 mg/ml Matrigel^®^ and delivered into SeedEZ scaffolds that were placed into PerfusionPal insert such that the culture medium was above and below the scaffold. The Blood Substitute was positioned underneath the culture medium, and the cultures were perfused.

**TABLE 2:** Abbreviations used to define experimental geometry.

ALI	Air-liquid interface, refers to the interface between the air and the culture medium.
LLI	Liquid-liquid interface, refers to the interface between the Blood Substitute and the culture medium.
Medium column	The total height of the medium column in a well which includes the SeedEZ scaffold thickness in 3D cell cultures.

For all studies, the controls had the following inadequacies:

• In the absence of multi-well plates having the same diameter as that of PerfusionPal insert, 2D Plate and 3D Plate control cultures had a larger well diameter than the PerfusionPal insert (3D Static and 3D Perfused cultures).
• For the same volume of medium used, the height of the medium column in PerfusionPal (3D Static and 3D Perfused) was larger than in the 2D Plate and the 3D Plate, thus reducing the benefit of PerfusionPal because larger columns of medium drastically limit cellular oxygen availability (Buck et al., 2014).
• In the 12-well PerfusionPal insert (3D Static and 3D Perfused), the medium column was 1.4 times larger than in the 24-well plate controls (2D Plate and 3D Plate).
• In the 48-well PerfusionPal insert (3D Static and 3D Perfused), the medium column was 2.8 times larger than in the 48-well plate controls (2D Plate and 3D Plate).

### 3D Cell Culturing in SeedEZ Extends and Augments Baseline CYP3A4 and CYP1A1 Activity

CYP3A4 and CYP1A1 enzyme activities were measured at the indicated time points (Figure 4). Two different media were used for culturing and assaying, serum-free HIM^TM^ and serum-containing *InVitro* GRO CP, with a rationale that serum-supplemented medium may have positive effects in long term culture. To adhere the cells to SeedEZ scaffolds, either a Poly-D-Lysine (PDL) coating was used [3D Plate (PDL)], or the cells were delivered in 8 mg/ml Matrigel^®^ (3D Plate). CYP3A4 activity in 2D cultures in collagen-coated plates [2D Plate (Cn)] was negligible after day 0 (Figure 4A). In the 3D Plate (PDL) and 3D Plate condition, the activity on day 0 was lower than in 2D, but higher on day 1, followed by a steady increase until day 7, albeit only with serum-supplemented medium for the 3D Plate (PDL) condition (Figures 4A–C). Starting with day 3, CYP3A4 activity in the 3D Plate condition with serum-supplemented medium exceeded the gold standard, 2D cell culture activity on day 0. CYP1A1 activity was low in all conditions on day 0 and day 1, and remained low in the 2D Plate and 3D Plate (PDL) conditions (Figures 4D,E). However, CYP1A1 activity in the 3D Plate condition with Matrigel^®^ began to rise around day 1 and remained substantially elevated (Figure 4F) with serum-supplemented medium. The use of Matrigel^®^ in combination with serum-containing medium resulted in higher CYP450 activities, surpassing 2D cell culture day 0 values from day 1 on (Figures 4C,F). It was also found that Matrigel^®^ and collagen did not contribute to the binding of drugs and assay reagents, but Poly-D-Lysine did (data not shown).

### 3D Cell Culturing With the Blood Substitute Increases Cellular Respiratory Metabolism in the 12-Well PerfusionPal Insert

To assess the effect of Blood Substitute (3D Static condition) and Blood Substitute and perfusion (3D Perfused condition) on cellular respiratory metabolism in 3D cell cultures, differentiated HepaRG cells and two cell lines, HepG2 and BT-474, were cultured in 3D Plate, 3D Static, and 3D Perfused conditions. The alamarBlue^®^ assay was run *in situ*. For 3D Perfused cultures, perfusion was halted during assaying and the assay run as in the 3D Static cultures. For each cell type, the results were normalized to their respective 3D Plate conditions. Despite the 1.4 times larger medium column in 3D Static and 3D Perfused cultures than in 3D Plate controls, the data revealed approximately a two-fold increase in cellular respiratory metabolism when cells were cultured in the presence of Blood Substitute (3D Static vs. 3D Plate) (Figure 5A). This was further enhanced by perfusion (3D Perfused vs. 3D Static) that had the most significant effect on respiratory metabolism in HepG2 cells with approximately threefold higher respiratory metabolism in the 3D Perfused vs. the 3D Plate condition.

### Culturing PHH, Differentiated HepaRG Cells, iPSC-Derived Hepatocytes, and HepG2 Cells With the Blood Substitute Consistently Increases CYP1A1 Activity

Hepatocytes from various sources were assayed for CYP1A1 activity to assess the effect of 3D cell culturing, Blood Substitute, and perfusion (Figure 5B). The assay was run *in situ*. For the 3D Perfused condition, perfusion was halted during assaying and the assay run as in the 3D Static condition. All cultures were assayed after 5–7 days in culture in the 2D Plate, 3D Plate, 3D Static and 3D Perfused conditions. The results were normalized to the respective 2D Plate condition for all cell types. 3D culturing with Matrigel^®^ in the SeedEZ scaffold (3D Plate condition) had no effect on CYP1A1 activity in HepG2, HepaRG, and iPSC-derived hepatocytes compared to their 2D counterparts (2D Plate). Although not significant, PHH 3D Plate activity was almost threefold higher than in 2D Plate. 3D Static culturing with the Blood Substitute enhanced CYP1A1 activity over 3D Plate culturing for all cell types. There was at least a twofold increase in CYP1A1 activity in 3D Static cultures relative to the respective 2D cell cultures, with the increase in activity for HepaRG and iPSCs being significant. 3D Perfused culturing (perfusion with the Blood Substitute) contributed to a further increase in CYP1A1 activity that was significantly higher than the 2D Plate and 3D Plate conditions for all cell types, but was the most pronounced for HepaRG cells. In PHH, the activity was over 50% higher with Blood Substitute and perfusion relative to the 3D Plate. Despite the 1.4 times larger medium column in 3D Static and 3D Perfused cultures than in 2D Plate and 3D Plate controls, the data shows that PerfusionPal increases CYP1A1 activity because of improved oxygenation and perfusion.

### Enhanced Oxygenation of HepG2 Cultures Using Perfused Blood Substitute

To demonstrate the utility of Blood Substitute to enhance extracellular oxygen availability, DO concentration in the culture medium was measured using a real-time oxygen consumption monitoring system (Lucid Scientific). Oxygen probes, inserted into custom lids, were positioned such that they were just above the top of 2D or 3D cultures (Figure 6A). The medium was not changed during studies to eliminate the spikes in oxygen tension after media changes. During measurements, the probes were moving 400 μm up and down to calculate the oxygen flux and the OCR. The experiments were carefully designed to isolate the effects of 3D culturing, Blood Substitute, and perfusion. For the 2D Plate/3D Plate study (Figures 6B,C), the culture medium for both conditions was above the cultures to isolate the effect of cellular distribution and the ECM on oxygen consumption. When comparing 3D Plate (U) to 3D Static (Figures 6D,E), the medium was both above and below the scaffold, but only the latter had the Blood Substitute underneath the medium to isolate the effect of the Blood Substitute on oxygen delivery to cells. Lastly, to examine the effect of perfusion, 3D Perfused was compared to 3D Static. The 3D Static condition corresponds to the fully infused 3D Perfused condition, i.e., the condition in which the level of medium above the scaffold is the highest.

In the experiment comparing 2D Plate and 3D Plate cultures, DO concentration for both showed an initial drop. The drop was more pronounced for 3D Plate than for 2D Plate, indicating that the cellular demands for oxygen immediately upon plating are higher in 3D than in 2D (Figure 6B). Correspondingly, the OCR slope in 3D Plate is steeper than in 2D Plate, corroborating higher oxygen consumption in 3D after plating (Figure 6C). After 24 h, DO concentration for 3D Plate had nearly reached a steady-state value of 50 μM. 2D Plate took nearly 96 h to reach its steady-state value of ∼70 μM. The corresponding OCR for the two conditions shows a higher steady-state demand for oxygen by the 3D cultures, despite oxygen diffusion limitations in the Matrigel^®^ matrix (Figure 6C). The peak OCR for 2D Plate and 3D Plate were 2.5 nmol/min/million cells and 4 nmol/min/million cells, respectively. The OCR (lit.), a dashed line in Figure 6C, refers to the value of OCR for HepG2 cells reported in the literature (Ehrlich et al., 2019).

Comparing 3D Plate (U) to 3D Plate, the rate of change in oxygen concentration due to cellular respiration after plating, although somewhat higher for 3D Plate (U) than 3D Plate is generally comparable (Figures 6B,D) (Note that the time scale is different in Figures 6B,D). The oxygen concentration in 3D Plate (U) did not reach a steady-state value until ∼120 h, and this value of 75 μM was similar to that in 3D Plate. This is due to the smaller column of medium above the scaffold in the U-bottom configuration, and therefore a greater sensitivity to evaporative losses which presumably extended the time to reach steady-state. Additionally, although many wells were sampled, only the data from the outer wells were included since the probes in the 3D Static and 3D Perfused conditions were in the outer wells. This provided the fairest comparison for this study, because oxygen probes could not have been integrated at the center due to technical limitations. Oxygen concentration in 3D Static also decreased after plating, but at a much slower rate than in 3D Plate (U), suggesting that the availability of oxygen to the cells was greater due to the LLI. Although comparable during the first 24 h, 3D Static cultures had significantly higher OCRs than 3D Plate (U) cultures later on.

The oxygen concentration for the 3D Perfused condition shows an expected sinusoidal pattern where the maxima correspond to the lowest position of the Blood Substitute (ALI closest to cultures), and the minima correspond to the highest position of the Blood Substitute (LLI closest to cultures). One perfusion cycle (2 mm down and 2 mm up) lasts 12 h. As shown in Figure 6D, the initial drop in oxygen concentration right after plating is practically negligible only in this condition. After 36 h, the 3D Perfused condition reached a steady-state with an average oxygen concentration of 175 μM. The peak concentrations of 200 μM were as high as the cell-free controls (not shown). The lowest concentrations (∼135 μM) were still higher than in any other condition. The pump was turned off at 96 h, stopping the sinusoidal pattern. The rise in values after this point corresponds to evaporation which was inevitable due to adaptation of the lid to accommodate oxygen probes. Due to evaporation, only the first 72 h of data are presented for OCR (Figure 6E). All of the 3D conditions showed a similar rise in OCR in the first 24 h. Beyond this time, 3D Plate (U) leveled off to a value of approximately 5 nmol/min/million cells. The OCR for the 3D Static and 3D Perfused conditions continued to rise. For an adequate comparison, 3D Perfused OCR values corresponding to the same volume of medium above the scaffold as in the 3D Static condition are shown. The maximum OCR for 3D Static was approximately 10 nmol/min/million cells. 3D Perfused had approximately the same values for the same volume of medium above the scaffold as in 3D Static.

### Metabolic Changes in HepG2 Cells in the Presence of Blood Substitute and Perfusion

We hypothesized that increased oxygen availability would enable cells to shift from anaerobic glycolysis to aerobic metabolic pathways in immortalized cell lines. To test this hypothesis, HepG2 cells were plated in 2D and 3D, with and without Blood Substitute and perfusion (Figure 7A) and cultured for 48 h in serum-, glucose-, and pyruvate-free medium as well as in media supplemented with combinations of glucose and pyruvate (Figure 7B). The maintenance medium for HepG2 cells contains serum, low glucose (5.5 mM), and pyruvate and was included for comparison. The alamarBlue^®^ assay was run *in situ*. For 3D Perfused cultures, perfusion was halted during assaying and the assay was run as in the 3D Static cultures. The conditions that included glucose but not pyruvate limited the ability of the cells to enter the citric acid cycle as the only source of pyruvate was as a product of glycolysis. The condition that lacked glucose, but included pyruvate essentially forced the cells to produce energy through aerobic pathways. The condition lacking both glucose and pyruvate was included to assess the ability of the cells to metabolize other carbon sources. The redox indicator alamarBlue^®^ was used as a reporter because resazurin is reduced to fluorescent resorufin by multiple metabolic pathways (Abe et al., 2002; Mommsen and Moon, 2005; Rampersad, 2012). When cultured in maintenance medium, moving from 2D to the 3D conditions increased metabolism with 3D Perfused resulting in significantly higher signal than both 2D Plate and 3D Plate. Without glucose or pyruvate, the metabolism was effectively zero for all conditions, although 3D Static and 3D Perfused fared marginally better. Adding glucose improved metabolic activity, with 3D Perfused having significantly higher activity than 2D Plate with low levels of glucose in the medium. Culturing with pyruvate without glucose improved metabolic activity slightly over having no energy source. Including low glucose along with the pyruvate significantly increased metabolic activity compared to pyruvate alone in 3D Static and 3D Perfused. Additionally, activity in 3D Perfused was significantly higher than both 2D Plate and 3D Plate. While not always statistically significant, 3D Static and 3D Perfused cultures had the highest metabolic activities for all media tested, except for high glucose medium, despite the 2.8 times larger medium column than in 2D Plate and 3D Plate controls.

It should be emphasized that the increased gas dissolving capacity of Blood Substitute is passive and driven by ambient availability. Hence, Blood Substitute cannot deliver more gas than is available in the cell culture incubator. However, Blood Substitute delivers more gas than would otherwise be available to cultures having solely apical exposure to the environment (ALI). The release of oxygen from the Blood Substitute is driven by the concentration gradient at the LLI, which depends on the cellular consumption. One concern with increased oxygen availability is the possibility of increased production of ROS, which could lead to inflammatory cell responses. To address the concern, HepG2 cells were grown in 3D in a multi-well plate or in PerfusionPal for 17 days, after which, ROS levels were assessed by H_2_DCFDA conversion to fluorescent DCF (Figure 7C). There was no difference between the two conditions, indicating no correlation between increased oxygen availability and ROS production. Additionally, glucose consumption on day 16 was lower in the 3D Perfused compared to the 3D Plate condition, suggesting that PerfusionPal contributes to less proliferative and more differentiated phenotypes even in cell lines (Figure 7D).

### Assay Limitations Transitioning From 2D to 3D Cell Culture

As underscored by assay manufacturers (PROMEGA, 2020), cell-based assays were originally designed and optimized for studying cells cultured in a monolayer or in a suspension. Such assay designs do not take into account the density and surface-to-volume ratio of 3D cell cultures that may limit cell lysis or overwhelm the assay chemistry. 3D cell cultures such as spheroids or hydrogel-based cultures pose diffusion limitations for assay reagents. Compared to a 2D monolayer of cells, 3D cell constructs are denser with a high number of cells in a small volume and/or often have differing diffusion kinetics through the ECM and/or the cellular mass. As outlined by PROMEGA (2020), “Standard protocols are not likely to work with 3D cultures if you need to isolate a protein or a metabolite from the cytoplasm. The protocol and reagent(s) must be optimized for 3D cultures.” For this reason, 3D cell cultures fail to outperform 2D in direct head-to-head comparison due to assay kinetics and diffusion challenges as shown later in this section. In certain instances, however, when 3D cell cultures overwhelmingly outcompete their 2D counterparts, we can see these differences as shown by alamarBlue^®^ assay in HepG2 cells *after 48-h long culturing* in 3D Plate, 3D Static, and 3D Perfused conditions (Figure 7B). Therefore, the enhancement of metabolic activity in 3D compared to 2D may actually have been greater than detected (Figure 7B) due to challenged assay kinetics in Matrigel^®^, and perhaps even greater considering approximately 12% slower doubling time in 3D vs. 2D (data not shown).

To elucidate the differences in readings in 3D vs. 2D, we tested alamarBlue^®^ and two CellTiter-Glo^®^ assays, one of which was optimized for 2D cell cultures (CellTiter-Glo^®^ 2.0) and the other for 3D cell cultures (CellTiter-Glo^®^ 3D). For the CellTiter-Glo^®^ assay, HepG2 cells were plated in a 48-well plate in 2D, in Matrigel^®^ without a SeedEZ scaffold, in a SeedEZ scaffold without Matrigel^®^, or in Matrigel^®^ in a SeedEZ scaffold. Cells were allowed to settle for 1 h, prior to initiating a CellTiter-Glo^®^ reaction for either 30 or 60 min, with or without shaking. The presence of Matrigel^®^ significantly reduced the CellTiter-Glo^®^ signal regardless of kit, reaction time, shaking, or the presence of SeedEZ (Figures 8A,B). The luminescence values for the CellTiter-Glo^®^ 3D assay kit were substantially higher than the CellTiter-Glo^®^ 2.0 kit, but the signal reduction was the same. Without Matrigel^®^, the signal was the same or higher in SeedEZ than in the 2D conditions in all cases. With the alamarBlue^®^ assay, the signal in 3D (cells seeded in Matrigel^®^ in a SeedEZ scaffold) was 75% lower than in 2D (Figure 8C). This was slightly mitigated by adding an agitation step at the end of the assay, but the signal reduction was still substantial.

AlamarBlue^®^, like the majority of cell-based assays yields much lower signal when working with 3D cell cultures than with 2D cell cultures. In contrast, CellTiter-Glo 3D assay is optimized for use with 3D cell cultures and yields higher signal when cells are seeded in Matrigel^®^ in the SeedEZ scaffold than does CellTiter-Glo^®^ 2.0 in 2D cell cultures. However, a direct comparison between 2D and 3D is still not feasible because CellTiter-Glo 3D has higher values when working with 2D cell cultures than with 3D cultures. Despite this caveat, we chose to compare all of our 3D assay results to 2D because we saw overwhelming improvements in 3D relative to 2D that were sufficiently high not to be “masked” by assay challenges in 3D. This was the case for assays run 48 h *after* culturing in 3D as shown in Figure 7B. However, it should be highlighted that the actual magnitude of improvements in 3D was higher than shown.

### Effect of Perfusion and the Blood Substitute on CYP450 Activity in Primary Human Hepatocytes

CYP450 activity was measured using luminescent CYP450-Glo assays or fluorescent substrates. PHH were plated in 2D in a 24-well plate or in 3D in a 12-well PerfusionPal insert and perfused. After 7 days in culture, the SeedEZ scaffolds were transferred to a 24-well plate and the luminescent and fluorescent CYP assays were multiplexed when possible and run in the absence of perfusion and Blood Substitute. It was reasoned that the transfer of scaffolds from PerfusionPal to a new multi-well plate for assaying would enhance comparative rigor considering the short duration of assays that could only capture a snapshot of the perfusion cycle. Since all assays were run as in 2D Plate, this allowed for a direct, head-to-head comparison of the effect of PerfusionPal culturing vs. 2D culturing. Results were normalized to baseline (uninduced) activity in 2D for each respective CYP. Despite a 1.4 times larger medium column in PerfusionPal than in 2D Plate controls during 7-day culturing, baseline activity in PerfusionPal was significantly increased for all CYP450 enzymes (Figure 9A). CYP1A1, 1A2, and 2C9 showed a 2 to 4-fold increase, 2C19 and 3A4 showed a 10-fold increase, and CYP2B6 showed a nearly 40-fold increase in activity. When cells were induced using FDA-approved inducers, PerfusionPal had significantly higher induction of all CYPs compared to the induction in 2D (Figure 9B). Additionally, for all but CYP1A1, the uninduced activity in PerfusionPal was higher than the induced activity in 2D which is considered the gold standard. When induced, CYP3A4 activity in PerfusionPal spiked to a nearly 25-fold increase relative to the baseline activity in 2D.

To isolate the effects of 3D culturing, Blood Substitute, and perfusion variables on CYP450 activity, the activity was measured for an expanded panel of plating and culturing conditions using the identical (Figures 10K,L) or similar (Figures 10A–J) volumes/media columns and flow rates as in the previously presented oxygen measurements in PerfusionPal (Figure 6, 3D Static and 3D Perfused). The assays were run after the transfer of the SeedEZ scaffolds from all 3D conditions (3D Plate, 3D Static, and 3D Perfused) into a 48-well plate containing the same volume of fresh medium as in 2D Plate for post-transfer assaying, and a direct comparison.

The culturing was still plagued by the 2.8 times larger medium column in the 48-PerfusionPal insert (3D Static and 3D Perfused cultures) than in the 48-well plate (2D Plate and 3D Plate controls) due to differences in well diameters. Considering that 3D cell cultures were superior to 2D despite diffusive assay limitations (Figures 5, 7), and that CYP450 activity in the 12-well PerfusionPal was superior despite a 1.4 times larger medium column than in 2D Plate controls (Figure 9), the study was run.

CYP450 activities were significantly elevated in all 3D cultures compared to 2D, if not by day 4, then by day 7 (Figure 10). Collagen coating had no effect on the activity in 2D, except for CYP1A2 on day 4 (Figure 10I). With the exception of CYP1A1 (Figures 10C,D), the activity in 3D cultures was unaltered or increased from day 4 to day 7. However, the attempt to isolate the variables was unsuccessful. Still, despite the 2.8 times larger medium column in PerfusionPal (3D Static and 3D Perfused cultures) than in 2D Plate and 3D Plate controls, the oxygen-dependent CYP450 activity did not deteriorate in PerfusionPal, and was still comparable to 3D Plate and 2D Plate controls, with CYP2B6 being an exception (Figures 10K,L).

### Effect of Perfusion on Albumin Production in PHH

Albumin production is an indicator of cell health and differentiation state. Levels of human albumin were measured in the media of the cultures in the previous CYP450 activity study after 4 and 7 days in culture (Figure 11). The data are reported as absorbance since there were issues with the calibration curves. Overall, as with the CYP450 data shown in Figure 10, 3D cultures did better than 2D, but the positive effects of superior oxygen delivery and perfusion were not substantial, and likely damped by the 2.8 times larger column of medium in the 48-well PerfusionPal insert than in the 48-well plate.

## Discussion

Cell culture models are invaluable research tools that have contributed to extraordinary medical and scientific discoveries. Remarkable advances with 3D cell cultures and 3D culture-based organ-on-a-chip technologies are opening the door to recapitulate complex aspects of human physiology, pathology and therapeutic responses (Low and Tagle, 2017; Tagle, 2019; Low et al., 2020). The challenges regarding throughput, assay multiplexing, and experimental complexity are increasingly addressed to ensure that organ-on-a-chip 3D models become a routine methodology accepted by academic and industrial stakeholders worldwide. To move the field forward, we presented a throughput-scalable organ-on-a-chip system, Perfused Organ Panel with PerfusionPal insert system, that requires a single tube to operate 48 statistically independent, 3D cell culture organ models. Then, we introduced in-well perfusion that eliminates the loss of cell signaling and drug metabolites in the otherwise one-way flow of perfusate. The cell secretome comprises autocrine and paracrine molecules, cytokine release, growth factors, neurotrophic factors, etc, which are critical for cell growth in three dimensions and virtually all cell functions. In PerfusionPal, the secretome and metabolome remain confined to the organ well to identify metabolites and biomarkers of disease and therapeutics response that are undiluted by a large dead volume of media in tubes and bottles that normally feed organs-on-a-chip. In-well perfusion renders PerfusionPal a zero-dead-volume fluidic device that: (1) provides a concentrated secretome and metabolome, (2) allows sampling of the medium using an ordinary micropipette in a familiar multi-well plate format, and (3) reduces cost of drugs and reagents that are wasted in microfluidic infrastructure (tubes and bottles) that support organ-on-a-chip devices. Next, to augment the physiological relevance of perfused 3D cell culture models, we tackled the problem of oxygen transport by blood *in vitro* using, for the first time, a synthetic hemoglobin analog, Blood Substitute, to improve delivery of respiratory gases to cells. *In vivo*, about 98% of oxygen delivery takes place via reversible binding to hemoglobin. Lena Biosciences’ Blood Substitute belongs to a class of breathable liquids with enormous gas carrying capacity. These molecules were extensively studied in liquid ventilation and liquid breathing due to their high solubility of respiratory gases, high density so that they descend through the lungs and open the areas of atelectasis, inertness, and the lack of metabolism, with complete elimination through exhalation or transpiration through the skin. This allowed static 3D cell cultures in PerfusionPal to “breathe” from their apical side via a medium-air interface, from their basal side via a medium-Blood-Substitute interface, and in perfused cultures via a gas-enriched medium perfused interstitially. Then, we showed that improved oxygenation has a beneficial effect even on cell lines by shifting metabolic pathways toward OXPHOS that contributes to the maintenance of differentiated phenotypes *in vitro*. Lastly, we showed that CYP450 activity of PHH cultured in 3D is increased and prolonged compared to the 2D cell culture gold standard with important ramifications for drug metabolism and disposition *in vitro*.

PerfusionPal is a unique and elegant platform for tissue and disease modeling. From the engineering perspective, this simple system allows parallel perfusion of 48 statistically independent organ cultures in their own media using only a single tube and a single pump. A comparable organ-on-a-chip solution would require 96 tubes, 48 pumps, and 48 media bottles. PerfusionPal is also an open system that integrates continuously ventilated perfusion to equilibrate both the Blood Substitute and the culture medium with respiratory gases. This eliminates the need for complex, large-scale equipment used in bioreactors to continuously charge PFC emulsions with 95% oxygen. Blood Substitute mimics the function of hemoglobin by delivering oxygen to parenchymal cells via the perfusing culture medium that models pericellular, interstitial flow. Using Blood Substitute underneath the medium in each culture well creates a second gas exchange interface on the basal side of 3D cell cultures to prevent cell ischemia and necrosis. Lastly, Blood Substitute serves as a piston that raises and lowers the medium in each well to achieve in-well perfusion that: (1) delivers O_2_, CO_2_, and nutrients to the 3D cultures more efficiently, (2) does not allow cross-talk between the wells, and (3) reduces the risk of culture contamination because the Blood Substitute does not support microbial growth and it is the only liquid in the tube used to perfuse all cultures.

Tuning the culture environment is critical for prolonging hepatocyte functions *in vitro*. The choice of coatings, hydrogels, and medium can have substantial effects on cell behavior in the SeedEZ scaffold (Figure 4). For example, a synthetic peptide, PDL-coating vs. Matrigel^®^ was used to isolate the effect of integrin signaling on CYP450 activity. 2D cultures in collagen-coated plates with serum-free medium showed good CYP3A4 activity on day 0, the day of plating. However, this activity quickly dropped off by day 1 and did not recover. Seeding the hepatocytes in Matrigel^®^ in SeedEZ scaffolds and using serum-containing medium resulted in ∼60% lower activity on day 0, followed by progressive enhancement over time that, by day 3, exceeded the 2D day 0 benchmark. Compared to spheroid cultures that require several days to 1 week to assemble and ensure uniform oxygen diffusion to the core (Bell et al., 2016; Berger et al., 2016), growing PHH in SeedEZ is advantageous because the CYP450 activity recovers faster, and the cells are at a lower risk of oxygen deprivation and necrosis. Therefore, 3D cell cultures in SeedEZ could be a useful tool for the assessment of metabolism of slow to metabolize drugs and less expensive and laborious to use compared to a relay assay (Di et al., 2012).

We hypothesized that the advantages of culturing cells in 3D in SeedEZ scaffolds would be further enhanced by the addition of the gas-carrying Blood Substitute and perfusion as more oxygen would be delivered to the cells in a manner analogous to hemoglobin-based oxygen delivery *in vivo*. Total cellular respiration in cell lines (HepG2 and BT-474) and in a terminally differentiated hepatocyte line (HepaRG) confirmed that 3D cultures were metabolically more active when Blood Substitute and perfusion were introduced, and in the case of the HepG2 cell line, when Blood Substitute was present even without perfusion (Figure 5A). Previous studies have shown that oxygen contributes to differentiation and phenotypic stability in HepaRG cells (van Wenum et al., 2018). Hyperoxia (40% atmospheric oxygen) significantly elevated common markers of liver function and CYP3A4 activity, while hypoxia (5% atmospheric oxygen) kept the cells in a proliferative state and inhibited differentiation. Similar effects were observed with HepG2 cells (van Wenum et al., 2018). Therefore, a plausible explanation for the increase in respiratory metabolism in differentiated HepaRG cells, and HepG2 and BT-474 cell lines cultured in PerfusionPal is a metabolic switch toward OXPHOS that yields 15-fold higher net ATP than glycolysis. This switch directs the cells toward a differentiated state with seemingly higher mitochondrial utilization and makes PerfusionPal useful for the assessment of mitochondrial liabilities.

The improvements in cellular function in response to Blood Substitute and perfusion extended to CYP1A1 activity in hepatocytes from various sources (Figure 5B). In PerfusionPal (3D Perfused), CYP1A1 activity was significantly increased in HepG2, HepaRG, iPSC-derived hepatocytes, and PHH compared to 2D and 3D Plate controls. HepaRG and iPSC-derived cells also exhibited this increase solely with the addition of Blood Substitute (3D Static). As the majority of CYP450 enzymes catalyze oxidation reactions, the data suggest that the Blood Substitute, with and without perfusion, increases the availability of oxygen *in vitro*. Previous studies demonstrated that hyperoxia (40–95% atmospheric oxygen) promotes liver specific functions and facilitates cellular adaptation to the *in vitro* environment suggesting that higher availability of DO may be able to prevent de-differentiation of primary hepatocytes *in vitro* (Poyck et al., 2008; Kidambi et al., 2009; Boulter et al., 2012). However, rather than artificially raising the atmospheric oxygen, Blood Substitute simply makes the oxygen more readily available to the cells in response to the oxygen gradient created by the cellular demand. This provides for an adaptive oxygen delivery method that quickly responds to the cellular metabolic needs in a manner analogous to the *in vivo* situation. Another important distinction between the Perfused Organ Panel environment and previously reported high atmospheric oxygen environments is that the likelihood for ROS damage is negligible. Perfused Organ Panel operates under normoxia in which DO cannot exceed ambient values because neither the Blood Substitute nor the medium can dissolve more than what is available in the incubator environment. Under hyperoxia, dissolved oxygen is, at a minimum, high at the air-liquid interface where the culture medium is equilibrated with the gaseous environment. Beyond certain thresholds, prohibitively high oxygen concentration could induce oxidative stress and formation of ROS that may ultimately lead to lipid peroxidation, protein damage, and cell apoptosis when cellular antioxidant capacity falls below the level of ROS production. The use of Blood Substitute and perfusion is therefore a safe alternative to high atmospheric oxygen environments to maintain liver specific functions.

The improved capacity of the PerfusionPal to deliver oxygen to 3D cell cultures of HepG2 cells was demonstrated using sensors measuring DO in the medium above the cultures. Studies revealed greater oxygen demand in 3D cultures compared to 2D with a correspondingly higher OCR in the steady-state of 4 nmol/min/million cells and 2.5 nmol/min/million cells, respectively. The latter is comparable to previously reported HepG2/C3A OCR value of 2 nmol/min/million cells (Ehrlich et al., 2019). Notably, although HepG2 cells have 12 times lower OCR than PHH (Ehrlich et al., 2019), we were able to detect significant differences when culturing HepG2 cells in 2D and 3D without and with perfusion and/or Blood Substitute. Despite seemingly higher oxygen diffusion limitations in Matrigel^®^ -filled SeedEZ (3D Plate) than in 2D Plate, oxygen concentrations rapidly fell after plating in 3D but not in 2D. This points to higher oxygen demands from the start of culturing with likely differences in metabolism early on when the cells are cultured in 3D. As the time progressed beyond 72 h, and glucose became depleted from the culture medium in the 2D Plate, the oxygen concentration reached its minimum. This suggests a likely shift in cellular metabolism toward OXPHOS when carbon sources became scarce in the 2D Plate.

3D Static relative to the comparably set up 3D Plate (U) that had the culture medium above and below the scaffold, reveals that Blood Substitute maintains higher oxygen concentration in the first 36 h, and especially, immediately after plating. The drop in DO during this period is much more gradual than it is in 3D Plate (U), suggesting that the Blood Substitute could be an invaluable, on-demand source of oxygen during the cellular adaptation to the *in vitro* environment when cellular oxygen needs are the highest. In PHH, OCR is 40–300% higher during the initial phase of cell attachment and within hours after plating than in steady culturing (Rotem et al., 1992; Balis et al., 1999). It has been shown that 95% oxygen culturing reduces this adaptation period during cell attachment and early culture when cellular OCRs are the highest (Kidambi et al., 2009). Studies further suggest that the reduction in the duration of the adaptation period correlates with the increase in metabolic competence early on, and that achieving high metabolism rates early on has important ramifications for downstream maintenance of prolonged metabolic functionality in primary cells (Kidambi et al., 2009). Mouse hepatocytes retained their functionality longer after isolation when cultured at 40% oxygen with OCRs slightly elevated from those found in the *in vivo* pericentral region of the mouse liver and over two times higher than those in the *in vivo* periportal region (Buck et al., 2014). This corroborates previous observations (Domansky et al., 2010) that the acceptable range of DO for *in vitro* perfusion may be higher than for the human *in vivo* periportal region, and acceptable within arterial blood oxygen concentration (104–146 μM) which is still below hyperoxic levels (Jungermann and Kietzmann, 2000). This provides additional evidence that oxygen is indeed critical for the *in vivo* to *in vitro* hepatocyte transition. Taken in perspective, higher oxygen concentration provided by the Blood Substitute at the initial stages of *in vitro* culture would seemingly facilitate adequate cellular adaptation to the *in vitro* environment and potentially contribute to the early establishment of high metabolic activity that, in turn, affects its long term maintenance. This assertion is supported by the OCR data (Figure 6). Specifically, the 3D Plate (U) condition leveled to a slightly higher OCR at 5 nmol/min/million cells compared to 4 nmol/min/million cells in the 3D Plate condition, suggesting that reducing the ALI distance improves oxygen availability, albeit only to an extent. The OCR in the 3D Static and 3D Perfused conditions all rose similarly to the 3D Plate (U) condition in the first 24 h, but when the 3D Plate (U) condition reached a plateau, the OCR values for the 3D Static and 3D Perfused conditions continued to rise until beginning to level off between 48 and 72 h at a peak OCR of 10 nmol/min/million cells. This continued OCR increase argues for the improved cellular oxygenation provided by the Blood Substitute. Note that for the 3D Perfused condition, only the OCR measurements that correspond to the fully infused state of perfusion, which is identical to the 3D Static condition in terms of medium column heights above and below the scaffold, are shown (Figure 6E) to adequately isolate the variables and directly compare the 3D Perfused to the 3D Static condition.

In PHH, energy production is highly dependent on OXPHOS. The cells contain more than 1,500 mitochondria that consume oxygen at high rates (Ehrlich et al., 2019). The sinusoids deliver oxygen at a rate of 72 nmol/min/million cells (Ehrlich et al., 2019). In human liver, the oxygen concentration reduces from ∼77 – 90 μM near the portal triad to ∼32 – 45 μM at the central vein (Jungermann and Kietzmann, 2000; Martinez et al., 2008; Ehrlich et al., 2019). Primary hepatocytes, appropriately cultured *in vitro*, consume oxygen at lower rates with the OCR ranging from 18 to 54 nmol/min/million cells (Ehrlich et al., 2019). The maximum OCR of HepG2 cells in our system was 60% of the lower end of this range. This is significant because HepG2 cells cultured in 2D are known to be glycolytic and de-differentiated. Growing them in the 3D Plate, 3D Plate (U), and in 3D Static and 3D Perfused conditions, gradually increased the cellular OCR from 2.5 nmol/min/million cells in 2D Plate, to 4 nmol/min/million cells in 3D Plate, to 5 nmol/min/million cells in 3D Plate (U), and lastly to 10 nmol/min/million cells in PerfusionPal. Furthermore, as shown by the oxygen concentration data, the 3D Perfused condition maintains a high level of DO, despite a highly elevated OCR. Although the oxygen concentration would certainly be lower when culturing PHH, the ability of the Blood Substitute to efficiently deliver oxygen may be able to successfully support the higher oxygen demands of PHH *in vitro*. This is because the Blood Substitute is constantly buffered with oxygen and the release of oxygen from the Blood Substitute is primarily driven by the oxygen concentration gradient in the culture medium that directly correlates with the cellular consumption (Ehrlich et al., 2019). In 3D Perfused cultures, the Perfused Organ Panel is designed to enable the medium column above the cultures to begin moving down when the perfusion starts. This effectively brings the ALI closer to the cultures and increases the pericellular oxygen concentration availability from the start when cells need it the most. At this point, we are unsure whether the LLI could potentially bring more oxygen to cells than can the ALI. The fact that HepG2 cells cultured with the Blood Substitute in the 3D Static condition ultimately had more than twice the OCR as compared with the 3D Plate (U) condition, suggests that the LLI could be more effective in delivery of oxygen than the ALI (as the Blood Substitute delivers DO between two liquid phases). Reduced CYP activity (Figure 10 vs. Figure 9) argues that larger columns of media reduce the availability of DO for primary cells in PerfusionPal, but perhaps not as drastically as observed previously (Buck et al., 2014), likely due to the Blood Substitute’s unique oxygen delivery method. Overall, the 3D Perfused condition provided the highest DO concentrations making PerfusionPal potentially ideal for the long-term maintenance of PHH without harmful effects of the ROS.

The increase in oxygen consumption was consistent with cell metabolism studies using alamarBlue^®^ as a reporter with media conditions designed to isolate aerobic and anaerobic metabolic pathways (Figure 7). As expected, the maintenance medium control fared the best as it was the only serum-containing condition. The condition lacking serum, glucose, and pyruvate demonstrated that, in a 48-h period, other carbon sources insignificantly contributed to the total metabolic activity. Compared with 2D Plate, the 3D Perfused condition had significantly higher metabolic activity in the low glucose condition, but not in the high glucose condition, suggesting, as expected, that mitochondrial metabolism may be more relevant to cells grown in 3D than in 2D and in culture systems with better cellular oxygenation. Providing pyruvate as the only readily available carbon source raised the metabolic activity for all culture conditions compared with the condition in which the cultures were given only DMEM. This condition effectively made the citric acid cycle and OXPHOS the only viable pathway for energy production. The trend suggests that the presence of Blood Substitute and perfusion improve mitochondrial metabolism, which is significant for drug testing in order to capture mitochondrial liabilities early on *in vitro*. The addition of low glucose showed a significant metabolism increase with perfusion. Of note are the comparisons between 3D Static and 3D Plate for all media conditions. For the two glucose-only conditions, the metabolic activity is nearly identical, but when pyruvate is present, there is a clear upward trend when Blood Substitute is added. This argues that Blood Substitute, likely through an increase in OCR, contributes to gains in the citric acid cycle. This increase becomes significant when perfusion is incorporated in the maintenance medium condition and in the low glucose/pyruvate condition. Collectively, the findings support the assertion that the presence of additional oxygen pushes cellular metabolism toward the aerobic pathway in PerfusionPal, especially considering that the medium column in 3D Static and 3D Perfused conditions was 2.8 times larger than in 2D and 3D Plate controls. Importantly, these improvements were not at the expense of cellular health because ROS remained comparable in 3D perfused cultures relative to 3D cultures in a multi-well plate (Figure 7C).

Consistent with the previous findings that oxygen drives differentiation of liver cell lines and maintains their phenotypic stability (van Wenum et al., 2018), enhanced cellular oxygenation in PerfusionPal likely contributed to more differentiated phenotypes but without the expense of ambient hyperoxia and the use of potentially toxic reagents, such as dimethyl sulfoxide to differentiate the cells (Ehrlich et al., 2019). Hence, Perfused Organ Panel may be able to allow better modeling of native tissues at a low cost by restoring *in vivo*-like functions even in cell lines, and in the context of primary cells, prevent their otherwise rapid de-differentiation *in vitro*.

To evaluate assay limitations when directly comparing 2D and 3D cell cultures, CellTiter-Glo^®^ and alamarBlue^®^ assays were carried out shortly after cell plating to eliminate differences in cell proliferation in 2D and 3D as a variable (Figure 8). We found that lytic CellTiter-Glo^®^ and metabolic alamarBlue^®^ assays were significantly lower in 3D than in 2D despite having the same number of cells. This indicates that although the data acquired show significant improvements with PerfusionPal, the full effects are dampened by diffusive limitations in 3D cell culture environments. It is important to differentiate that the studies assessing diffusion limitations (Figure 8) were carried out 1 h after plating, while the metabolic studies (Figure 7) were carried out after 48 h, when the cells had been able to adapt to their respective culturing environments.

Culturing PHH in PerfusionPal augmented and prolonged CYP450 activity. These improvements were captured despite having a 1.4 times larger medium column in the 12-well PerfusionPal insert than in the 24-well plate, 2D controls. In PerfusionPal (Figure 9), baseline activity was significantly increased for all assayed CYP450 enzymes. Most notably, CYP3A4 was increased more than 10-fold. CYP3A4 is responsible for the metabolism of more than 50% of drugs, and its induced activity, using an FDA-approved CYP450 inducer, had a nearly 25-fold increase over uninduced PHH cultured in 2D.

This study was expanded in 48-well PerfusionPal inserts to isolate specific variables that contributed to the previous findings. Unfortunately, due to a 2.8 times larger column of medium in the 48-well PerfusionPal insert versus the 48-well plate, we were not able to do so. Nonetheless, the study underscored two findings. First, it showed that even with such a considerable and unfavorable medium column bias, the CYP450 activity in PerfusionPal (3D Static and 3D Perfused) did not severely deteriorate, but rather stayed comparable to that in the multi-well plate (3D Plate). Second, the study demonstrated that culturing PHH in 3D in SeedEZ maintains a reasonable level of CYP450 activity. It is unknown whether encouraging improvements with Matrigel^®^ can be attributed solely to the integrin signaling (Moghe et al., 1996), trophic or other factors present in Matrigel^®^ or both. However, as with all hydrogels, Matrigel^®^ shape is difficult to control. This results in inconsistent formation of Matrigel^®^ culture drops or meniscus cultures having significant assay variability. In contrast, when cells are seeded in Matrigel^®^ into the SeedEZ scaffolds, their shape is consistent, cell distribution reproducible, and the cultures do not peel off the dish.

Increasing medium column height has drastic effects on pericellular DO concentrations. Studies conducted at 20% atmospheric oxygen showed a ∼75% reduction in pericellular DO in primary mouse hepatocytes cultured in a 2.6 mm versus a 1.3 mm medium column on day 1 (Buck et al., 2014). Addition of Matrigel^®^ resulted in a ∼90% decrease in a 2.6 mm medium column vs. a 1.3 mm medium column. Over the course of 4 days, cells cultured in a 2.6 mm medium column had over 50% reduced albumin production (with or without Matrigel^®^) compared to cells cultured in a 1.3 mm column of medium. In PerfusionPal (3D Static and 3D Perfused), despite a 2.8 times larger medium column than in 2D Plate and 3D Plate, albumin measurements did not show such a severe decrease (Figure 11). On day 4, albumin concentration was similar or elevated, and by day 7, higher than in controls. This is consistent with comparable CYP450 activity (Figure 10) under such a considerable column height disadvantage. Collectively, our studies argue that Blood Substitute and the LLI may be more efficient in oxygen delivery than the ALI, and that creating physiologically closer environments augments and extends CYP450 activity even in inherently variable PHH.

Many factors can upregulate CYP450 activity *in vitro* including exogenous inducers of Wnt/β-catenin signaling (Briolotti et al., 2015), chromatin remodelers (Isom et al., 1985; Henkens et al., 2007), culture media, and culturing methods. Culturing methods include co-cultures of PHH with non-parenchymal cells, use of ECMs, collagen sandwich, spheroid models, microfluidic organ-on-a-chip devices, bioreactors, hyperoxic environments, and their combinations. SeedEZ scaffold and Perfused Organ Panel can be a viable alternative without resorting to substantial, artificial manipulations to enhance CYP450 activity. An inert hemoglobin analog in the form of the Blood Substitute, provides the cells with an oxygen availability that is more physiologically representative and contributes to cellular differentiation. Reducing artificial manipulations required to maintain differentiated phenotypes *in vitro*, produces, in turn, a system that is seemingly more predictive of the *in vivo* tissue response.

In sum, PerfusionPal organ-on-a-chip insert system contributes to the growing field of predictive tissue models for disease modeling and drug testing *in vitro*, and pushes the envelope of *in vitro* research by enabling, for the first time, oxygen delivery to perfused and unperfused 3D cell cultures via a breathable hemoglobin analog. Specifically, we showed improvements in oxygen delivery to cells. Next, we showed that the aerobic cell metabolism was significantly enhanced without inflammation. Lastly, we showed that CYP450 enzyme activity in PHH was increased and maintained over time which validates the system as a suitable model for enhanced drug metabolism studies *in vitro*.

## Data Availability Statement

The raw data supporting the conclusions of this article will be made available by the authors, without undue reservation.

## Author Contributions

JS designed the research tools, designed the studies, performed the research, interpreted the data, and wrote the manuscript. WZ and SA performed the research, interpreted the data, and wrote the manuscript. RB and SI provided the research tools, performed the research, and interpreted the data. JV conceived the project, designed the research tools, designed the studies, performed the research, interpreted the data, and wrote the manuscript. All the authors contributed to the article and approved the submitted version.

## Conflict of Interest

PerfusionPal insert system, Perfused Organ Panel (PerfusionPal starter system), and SeedEZ scaffold are commercial products sold by Lena Biosciences. JS is the Chief Science Officer at Lena Biosciences and holds stock options of the company. JV is the President and Chief Executive Officer at Lena Biosciences and holds shares of the company. Lucid Scientific manufactures and sells oxygen analysis equipment. RB and SI are employees and stockholders of Lucid Scientific Inc. The remaining authors declare that the research was conducted in the absence of any commercial or financial relationships that could be construed as a potential conflict of interest.
